# Targeting NUFIP1 Suppresses Growth and Induces Senescence of Colorectal Cancer Cells

**DOI:** 10.3389/fonc.2021.681425

**Published:** 2021-07-23

**Authors:** Aling Shen, Meizhu Wu, Liya Liu, Youqin Chen, Xiaoping Chen, Mingkai Zhuang, Qiurong Xie, Ying Cheng, Jiapeng Li, Zhiqing Shen, Lihui Wei, Jianfeng Chu, Thomas J. Sferra, Xiuli Zhang, Nanhui Xu, Li Li, Jun Peng, Fenglin Chen

**Affiliations:** ^1^ Academy of Integrative Medicine, Fujian University of Traditional Chinese Medicine, Fuzhou, China; ^2^ Fujian Key Laboratory of Integrative Medicine on Geriatrics, Fujian University of Traditional Chinese Medicine, Fuzhou, China; ^3^ Department of Pediatrics, Case Western Reserve University School of Medicine, Rainbow Babies and Children’s Hospital, Cleveland, OH, United States; ^4^ Department of Gastroenterology, Fujian Medical University Union Hospital, Fuzhou, China; ^5^ Department of Physical Education, Fujian University of Traditional Chinese Medicine, Fuzhou, China; ^6^ Department of Health Management, Fujian Provincial Hospital, Shengli Clinical College of Fujian Medical University, Fuzhou, China

**Keywords:** NUFIP1 knockdown suppresses tumor growth, colorectal cancer, growth, senescence, ursolic acid

## Abstract

NUFIP1 is an RNA-binding protein that interacts with fragile X mental retardation protein (FMRP) in the messenger ribonucleoprotein particle (mRNP). We previously showed that NUFIP1 was upregulated in colorectal cancer (CRC), but how the protein may contribute to the disease and patient prognosis is unknown. Here we combine database analysis, microarray, quantitative PCR, and immunohistochemistry of patients’ samples to confirm our previous findings on NUFIP1 overexpression in CRC, and to reveal that increased expression of NUFIP1 in CRC tissues correlated with worse overall, recurrence-free, event-free and disease-free survival in patients, as well as with more advanced CRC clinicopathological stage. Loss of function analysis demonstrated that NUFIP1 knockdown suppressed cell growth *in vitro* and *in vivo*, inhibited cell viability and survival, and induced cell cycle arrest and apoptosis *in vitro*, as well as up-regulated Bax and down-regulated Bcl-2 protein expression. In addition, as a natural anticancer triterpene from various fruits and vegetables, ursolic acid (UA) treatment suppressed cell proliferation, down-regulated NUFIP1 protein expression, and further enhanced the effects of NUFIP1 knockdown in CRC cells *in vitro*. NUFIP1 knockdown up-regulated the expression of 136 proteins, down-regulated the expression of 41 proteins, and enriched multiple signaling pathways including the senescence-associated heterochromatin foci (SAHF) pathway. Furthermore, NUFIP1 knockdown enhanced the expression of senescence-associated-β-galactosidase (SA-β-gal), the SAHF markers HP1γ and trimethylation (H3k9me3), and the senescence-related protein HMGA2, as well as both p53 and its downstream p21 protein expression. Our findings suggest that NUFIP1 is overexpressed in CRC and correlates with disease progression and poor patient survival. NUFIP1 may exert oncogenic effects partly by altering senescence. UA may show potential to treat CRC by down-regulating NUFIP1.

## Introduction

Colorectal cancer (CRC) is the third most commonly diagnosed cancer type and the fourth leading cause of cancer mortality worldwide (1). The incidence and mortality of CRC are increasing in China (2), and2.2 million new cases of CRC and 1.1 million deaths are predicted by 2030 in the world (3). Although many breakthroughs in the diagnosis and treatment of CRC have been made over the past few decades, CRC-related mortality remains high (4). The progression of CRC is a multi-step process involving the deregulation of several oncogenes and tumor suppressor genes, which might be used as diagnostic and therapeutic targets (5). However, the precise mechanisms are poorly understood and novel diagnostic biomarkers need to be discovered (6). Molecular mechanisms involved in the occurrence and development of CRC should be studied in depth to improve its early diagnosis and help predict relevant treatment targets.

In a previous study to identify potential oncogenes in CRC, our team found that nuclear fragile X mental retardation-interacting protein 1 (NUFIP1) mRNA expression was up-regulated in CRC tissues compared with noncancerous colorectal tissues, while NUFIP1 knockdown suppressed CRC cell growth *in vitro*. This suggested the oncogenic potential of NUFIP1 in CRC. However, the clinical significance, biological function and underlying mechanism of NUFIP1 on CRC cell growth has yet to be investigated, which encouraged us to further explore the role of this RNA-binding protein in CRC.

NUFIP1 is a 495-amino-acid protein containing two C2H2 zinc finger motifs and one putative nuclear localization sequence (NLS) (7, 8). Early studies identified NUFIP1 as an RNA-binding protein in the mRNP particle associated with fragile X mental retardation. This particle is abundant in neuromas of cortex, hippocampus and cerebellum, and it interacts with FMRP (7) and FXR2P (9) to contribute to fragile X syndrome. Moreover, NUFIP1, in addition to roles in diseases such as fragile X syndrome and cancers, is involved in numerous cellular processes and responses to various stresses (10–17). Homozygous loss of NUFIP1 may contribute to psychomotor delays (18), and NUFIP1 gene polymorphisms have been associated with osteoporosis and obesity-related traits (19). Mutations in NUFIP1 have been detected in neuroblastoma (20), and a fusion of NUFIP1 with ETV-6 has been identified in acute lymphoblastic leukemia (21). However, the roles of NUFIP1 in solid tumors, including CRC, have not been evaluated.

In the current study, we combined microarrays, online database searches, cDNA array-based quantitative PCR (qPCR) and tissue microarray (TMA)-based immunohistochemistry (IHC) to analyze the expression of NUFIP1 in CRC samples and to correlate its expression with disease progression and prognosis. We also used *in vitro* and *in vivo* assays to investigate the effect of NUFIP1 knockdown on tumor growth. We explored the underlying mechanisms using proteomics, western blotting and immunofluorescence. Moreover, we investigated the potential of NUFIP1 to be a therapeutic target in CRC by treating cancer cells with UA.

## Materials and Methods

Human and animal procedures in this study were approved by the Fujian University of Traditional Chinese Medicine (Fujian, China). Animal procedures were conducted in accordance with the guidelines of the Animal Committee of the University.

### Bioinformatic Analysis

In a previous study, we screened DEGs on 14 pairs of CRC primary lesions and surrounding non-cancerous tissues (GEO submission: GSE113513) (22). We selected 10 of these genes and examined their expression in the GEPIA database (http://gepia.cancer-pku.cn/). Data were extracted from this database to examine association of NUFIP1 expression with clinicopathological stage of CRC and DFS.

The R2 application (http://r2.amc.nl) was used to explore the correlation between NUFIP1 expression and RFS of CRC patients using the log-rank method and the data in the dataset “Tumor Colon-CIT (Combat)-Marisa-566 rma-u133 p2; Tumor Colon (Core-Exon) Sveen-333 rma_sketvh-huex10p”.

### Antibodies and Reagents

Antibodies against HP1γ β-actin, H3k9me3 and HMGA2, p21and Bax, and the Senescence β-Galactosidase Staining Kit were purchased from Cell Signaling Technology (CST; Beverly, MA, USA). Anti-Bcl-2 antibody was purchased from Proteintech (Suite 300 Rosemont, IL, USA). Anti-p53 antibody was purchased from MBL (Minato-ku, Tokyo, Japan). Anti-NUFIP1 antibody, fetal bovine serum (FBS), trypsin-EDTA (0.25%), Pierce ™ BCA Protein Assay kit, and FxCycleTMPI/RNase Staining Solution were purchased from Thermo Fisher Scientific (Carlsbad, CA, USA). The Annexin V-AbFluor™ 647 Apoptosis Detection kit and antibody against glyceraldehyde 3-phosphate dehydrogenase (GAPDH) were obtained from Abbkine (Wuhan, Hubei, China). UA was purchased from Millipore Sigma (Billerica, MA, USA).

### Cell Lines and Cell Culture

Human CRC cell lines (HCT116 and HT-29 cells) were purchased from the Cell Bank of the Shanghai Institutes for Biological Science of the Chinese Academy of Sciences (Shanghai, China) and routinely cultured in RPMI1640 (Thermo Fisher Scientific; Carlsbad, CA, USA) or McCoy’s 5A medium (KeyGEN; Jiangsu, China) supplemented with 10% FBS. All cells were maintained in a humidified incubator at 37°C and 5% carbon dioxide. During long-term use of HCT116 and HT-29 cells, cells were checked using short tandem repeat genotyping and examined for mycoplasma contamination using real-time PCR.

### Lentiviral Transduction and Cell Growth Analysis

Lentivirus encoding control shRNA or shRNAs targeting the 10 DEGs were constructed by Shanghai GeneChem (Shanghai, China). HCT116 were seeded in 12-well plates for 16 h prior to viral infection, then transduced with lentivirus at a multiplicity of infection of 10 in cell culture medium for 6-8 h, and then were changed into fresh medium and culture for total 72 h. Transduced cells were reseeded into 96-well plates at a density of 2,000 cells/well in 100 μL of completed medium. Cell growth was monitored every day for five days using the Pathway 855 high-content image analysis platform (BD Biosciences, San Jose, CA, USA), and cells were counted.

### Quantitative PCR Analysis

Total RNA was extracted from cell line samples using TRIZOL (Thermo Fisher Scientific; Carlsbad, CA, USA) and converted to cDNA using reverse transcription-PCR (Thermo Fisher Scientific; Carlsbad, CA, USA). The resulting cDNA, or a commercial tissue cDNA array (Shanghai Outdo Biotech; Shanghai, China), was used to measure levels of NUFIP1 mRNA using an ABI 7500 Fast Real-Time PCR System (Applied Biosystems, Foster City, CA, USA) and the SYBR Premix Ex Tag (Thermo Fisher Scientific; Carlsbad, CA, USA). The following cycling conditions were used: 10 min at 95°C, and 40 cycles of 15 s at 95°C and 1 min at 60°C. Levels were quantified using the comparative Ct method and normalized to levels of GAPDH mRNA. The sequences of primers are listed in **Supplementary Table S2**.

### TMA and Survival Analysis

TMA slides of CRC tissue samples and their corresponding noncancerous tissues were obtained from Shanghai Outdo Biotech (cat. no. HColA180Su15, Shanghai, China). All pathology specimens were collected, along with complete clinical and pathologic data. The TMA was incubated with antibody against NUFIP1 (1:1000) using standard techniques. Antibody binding was captured using a Nano Zoomer 2.0 HT slide scanner (Hamamatsu Photonics) and processed using Nano Zoomer Digital Pathology View 1.6 software. The intensity and extent of IHC staining were assessed independently by two experienced pathologists blinded to the clinical and pathologic data. Staining intensity was assessed using a four-point scale (0, undetectable; 1, weak; 2, moderate; 3, strong), while the percentage of positively stained cells was expressed as one of four categories (0–25%, 26–50%, 51–75%, and 76–100%). The two scores were multiplied together to yield the final overall NUFIP1 score.

Based on the overall scores from the TMA slides, patients were assigned to groups showing high NUFIP1 expression (score 9-12) or low expression (score 0–8). The relationship between NUFIP1 expression and overall survival was evaluated using Kaplan-Meier analysis and assessed for significance using the log-rank test.

### Western Blot Analysis

Total proteins were extracted from cells using lysis buffer (Beyotime Biotechnology; Jiangsu, China) supplemented with protease inhibitor. The protein concentration was measured with BCA Protein Assay Kit (Thermo Fisher Scientific; Carlsbad, CA, USA), and equal amounts of protein were subjected to sodium dodecyl sulfate-polyacrylamide gel electrophoresis (10%). Proteins were then transferred onto polyvinylidene fluoride membranes. After blocking with blocking buffer (Beyotime Biotechnology; Jiangsu, China) for 2 h, the membranes were incubated with a primary antibody overnight at 4°C. The membranes were washed with TBST buffer, followed by incubation with the secondary antibody conjugated to horseradish peroxidase. GAPDH was used as loading control. Protein bands were detected with a chemiluminescence kit (Thermo Fisher Scientific; Carlsbad, CA, USA) and analyzed using the ImageLab software.

### Cell Confluence and Cell Counting

Cell confluence was observed by microscope (Leica Microsystems; Wetzlar, Germany) at 200× magnification. Then the cells were stained using 0.4% trypan blue, and analyzed using a Countstar Automated Cell Counter (Shanghai, China).

### *In Vivo* Experiments

Male nude mice (4-6 weeks old, 22-24 g) were purchased from Shanghai SLAC Laboratory Animal Company (Shanghai, China) and maintained in a special pathogen-free facility. Transduced HCT116 or HT-29 cells (1 × 10^6^) in 100 μL of M5A medium containing 50% Matrigel (BD Pharmingen; Franklin Lake, NJ, USA) were injected subcutaneously into the flank of nude mice (n=6). Tumor volume was measured using a standard caliper once every other day for 19 days, starting from the third day after first injection. The tumor volume was calculated using the formula: (length × width^2^)/2.

Mice were anesthetized with isoflurane and analyzed using an IVIS whole-animal imaging system (PerkinElmer; Santa Clara, CA, USA). Then mice were sacrificed, and tumor tissues were collected and weighed.

### CCK-8 Assay

HCT116 or HT-29 cells were transduced with sh-NUFIP1 or sh-Ctrl and treated (or not) with 10 μM UA. At the indicated time points, cell viability was measured by adding 10 μL Cell Counting Kit-8 (Abbkine; Wuhan, Hubei, China) into 100 μL medium per well. The cells were cultured for an additional 2 h at 37°C, and the absorbance was measured at 450 nm using a microplate reader (Thermo Fisher Scientific; Carlsbad, CA, USA).

### Colony Formation Assay

Cell survival was analyzed by colony formation assay. HCT116 or HT-29 cells were reseeded into 12-well plates (500 cells/plate) and incubated for about 10-12 days at 37°C in a humidified atmosphere containing 5% carbon dioxide. Culture medium was changed every three days. At the end of experiment, the cells were fixed with 4% paraformaldehyde for 20 min, then stained with 0.01% crystal violet for 15 min. Colonies were counted and photographed.

### Cell Cycle Analysis

Cell cycle progression was analyzed using an FxCycle™PI/RNase Staining Solution in accordance with the manufacturer’s instructions. Briefly, cells were seeded into six-well plates, incubated for an additional 72 h, collected, and fixed with 70% cold ethanol (4°C) for about 24 h. Washed cells were incubated with a mixture of FxCycle™PI/RNase Staining Solution for 40 min at room temperature in the dark, then analyzed by flow cytometry (FACS Caliber; Becton Dickinson, San Jose, CA, USA).

### Cell Apoptosis Analysis

Apoptotic status was determined using annexin-V staining using Annexin-V-AbFlour™647 Apoptosis detection Kit (Abbkine; Wuhan, Hubei, China) with or without propidium iodide staining, followed by flow cytometric analysis. Briefly, cells were transduced with shRNA-encoding lentivirus, treated or not with UA, collected and incubated with the solution from the apoptosis detection kit. The percentage of apoptosis was analyzed by flow cytometry on a FACS Caliber (Becton Dickinson, San Jose, CA, USA).

### iTRAQ-Based Quantitative Proteomic Study and Pathway Analysis

Proteins were identified and quantified by secondary mass spectrometry using Thermo Scientific’s Q Exactive mass spectrometer (Thermo Fisher Scientific; Carlsbad, CA, USA). DEPs were identified based on the following criteria: │fold change│ in protein expression ≥1.5 and *P<*0.05.

KEGG (http://www.genome.jp/kegg) analysis was performed to analyze pathway enrichment in DEPs, while gene ontology (GO) (http://www.geneontology.org) was used to analyze signal transduction pathways. Enrichment of KEGG pathway and GO was considered significant when *P <* 0.05 (Fisher’s exact test).

### Senescence β-Galactosidase Staining

Transduced cells were washed with PBS and fixed with 1× fixing solution. After 10-15 min at room temperature, fixed cells were washed and incubated with β-galactosidase staining solution at 37°C overnight in a dry incubator. Images of β-galactosidase staining were taken using a microscopy (Leica; Wetzlar, German) at magnification of 400×.

### Immunofluorescence Staining

Immunofluorescence staining was performed following standard protocols. Cells were washed with PBS, fixed in 4% paraformaldehyde and permeabilized with 0.1% Triton-X-100. After blocking in 1% BSA, cells were incubated with diluted primary antibody (1:200) overnight at 4°C. After washing with PBS, cells were subjected to the secondary antibody for 1 h at room temperature, followed by Hoechst staining (Beyotime Biotechnology; Jiangsu, China). All images were captured using microscopy (Leica; Wetzlar, German) at magnification of 400×.

### Statistical Analysis

Statistical analysis was performed using SPSS 26.0 software (IBM, Armonk, NY, USA). Data were presented as mean ± standard deviation. Differences between two groups were assessed using the independent Student’s t test, and differences among three or more groups were assessed using one-way ANOVA. Kaplan–Meier survival differences were assessed using the log-rank test. *P* < 0.05 was considered significant.

## Results

### Identification of NUFIP1 as a Potential Target in CRC

**Figure 1:**
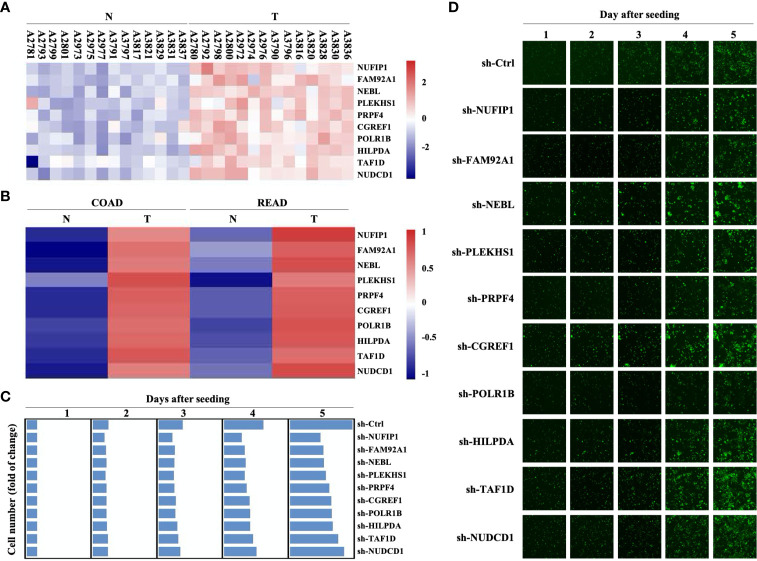
Gene expression profiling and high content screening suggest that NUFIP1 has carcinogenic effects. cDNA microarray analysis was performed to identify differentially expressed genes (DEGs) between 14 pairs of colorectal cancer (CRC) tissues (T) and adjacent normal tissues (N). High-content screening and lentivirus-delivered, shRNA-based interference was used to assess the effects of candidate genes on CRC cell growth. **(A)** Heatmap of the 10 selected DEGs. RKO cells were transduced with lentivirus encoding shRNAs against these DEGs, and cell growth was measured using multiparametric high-content screening. **(B)** NUFIP1 mRNA expression in CRC primary lesions (T) and non-cancerous tissues (N). **(C, D)** Effects of knocking downNUFIP1, FAM92A1, NEBL, PLEKHS1, PRPF4, CGREF1, POLR1B, HILPDA, TAF1D, or NUDCD1 on the growth of HCT116 cells. **(C)** Heatmap showing the growth of HCT116 cells. Data were normalized to cell number on day 1 and are represented as fold change. **(D)** Representative images of RKO cell growth. Image magnification at 40×.

A previous screening of differentially expressed genes (DEGs) on 14 pairs of CRC primary lesions and surrounding non-cancerous tissues identified 778 up-regulated and 1090 down-regulated genes (GEO Submission: GSE113513) (22). We decided to focus on 10 up-regulated genes that have not yet been extensively investigated as potential oncogenes (**Figure 1A** and **Supplementary Table S1**). Analysis in the GEPIA (Gene Expression Profiling Interactive Analysis) database (http://gepia.cancer-pku.cn/) also revealed significant up-regulation of these 10 DEGs in both colon adenocarcinoma and rectum adenocarcinoma, compared with non-cancerous colorectal tissues (**Figure 1B**). Transducing of Human CRC cells (HCT116 cells) with small hairpin RNA (shRNA) targeting NUFIP1, FAM92A1, NEBL, PLEKHS1, PRPF4, CGREF1, POLR1B, HILPDA, TAF1D, or NUDCD1 attenuated growth. These results confirmed the oncogenic potential of NUFIP1 in CRC (**Figures 1C, D**).

### NUFIP1 Is Highly Expressed in CRC Tissues

**Figure 2:**
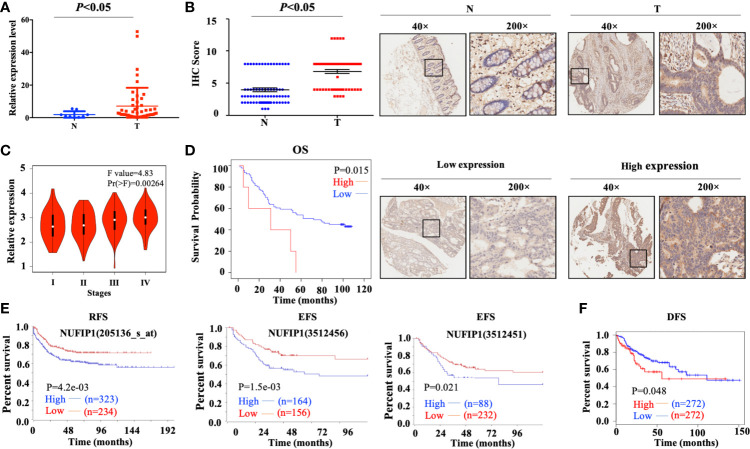
Levels of NUFIP1 mRNA and protein in colorectal cancer (CRC) tissues. **(A)** NUFIP1 mRNA expression in tumor tissue (T) and non-cancerous tissues (N) on the CRC cDNA chip, analyzed by qPCR. GAPDH was used as an internal control. P < 0.05 *vs.* normal tissue. **(B)** NUFIP1 protein expression in CRC tissues (T) and non-cancerous breast tissues (N), determined by immunohistochemistry of a tissue microarray. Representative images were taken at magnification of 40× or 200× (right panel); the final score was calculated as intensity score × percentage score (left panel, see Methods). P < 0.05 *vs.* normal tissue. **(C)** CRC clinicopathological stages according to the GEPIA database. **(D)** Kaplan-Meier plots of survival of CRC patients, stratified by NUFIP1 protein expression based on immunohistochemistry of a tissue microarray. (P < 0.05). Correlation between NUFIP1 mRNA expression and RFS, EFS, and DFS of CRC patients according to **(E)** the R2 application and **(F)** GEPIA database (P < 0.05). Survival was analyzed with log-rank test.

Quantitative PCR analysis on a CRC cDNA chip (Shanghai Outdo Biotech, Shanghai, China) in 15 pairs of CRC patients’ samples confirmed that NUFIP1 mRNA expression was up-regulated in CRC tissues, compared with matched noncancerous colorectal tissues (**Figure 2A**; P < 0.05, tumor *vs.* normal tissue; **Supplementary Table S3**). Verification of NUFIP1 protein expression by IHC analysis in CRC TMA (including 71 pairs of CRC samples) indicated that NUFIP1 was expressed in CRC tissues at higher levels than in matched noncancerous tissue (**Figure 2B**; P < 0.05, tumor *vs.* normal tissue).

### High Expression of NUFIP1 in CRC Correlates With Poor Prognosis

We further assessed the correlation between NUFIP1 expression and clinicopathological stages of CRC patients using the GEPIA database, and we found NUFIP1 expression to be higher in patients with more advanced CRC than in those with early CRC (**Figure 2C**). Higher NUFIP1 expression also correlated with shorter overall survival (OS) of CRC patients (**Figure 2D**; P < 0.05; **Supplementary Table S4**). Using the R2 application and GEPIA dataset, we found that higher expression of NUFIP1 correlated with shorter recurrence-free survival (RFS), event-free survival (EFS; **Figure 2E**; P < 0.05), and disease-free survival (DFS; **Figure 2F**; P < 0.05).

### NUFIP1 Knockdown Suppresses CRC Cell Growth *In Vitro* and *In Vivo*

**Figure 3:**
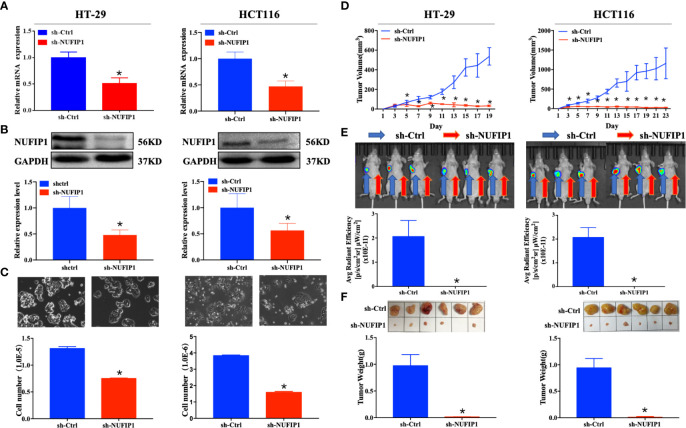
NUFIP1 knockdown inhibits colorectal cancer (CRC) cell growth. **(A–C)** HT-29 and HCT116 cells were transduced with lentivirus encoding either anti-NUFIP1 small hairpin RNA (sh-NUFIP1) or control shRNA (sh-Ctrl). **(A)** Knockdown of NUFIP1 in HT-29 and HCT116 cells was confirmed by qPCR. GAPDH was used as an internal control. *P < 0.05 *vs.* sh-Ctrl lentivirus. **(B)** Western blot showing the levels of NUFIP1 protein in HT-29 and HCT116 cells. NUFIP1 bands were quantitated using ImageJ software and normalized to GAPDH. *P < 0.05 *vs.* sh-Ctrl lentivirus. **(C)** Growth of HT-29 and HCT116 cells after knockdown of NUFIP1 by phase-contrast (light) microscopy at a magnification of 200×. *P < 0.05 *vs.* sh-Ctrl lentivirus. **(D–F)** A xenograft nude mouse model was established to explore the effect of NUFIP1 knockdown on tumor growth in HT-29 and HCT116 cells. A total of 1.0 × 10^6^ cells were injected subcutaneously into the armpits of BALB/c mice; these cells had been transduced with lentivirus encoding sh-Ctrl (left armpit) or sh-NUFIP1 (right armpit). The volumes **(D)**, fluorescence **(E)** and weights **(F)** of tumors from each mouse were determined and recorded. *P < 0.05 *vs.* sh-Ctrl lentivirus. All experiments were performed in triplicate.

The interference efficiency of NUFIP1 shRNA was validated at both mRNA and protein levels (**Figure 3A, B**; *P < 0.05 *vs.* sh-Ctrl lentivirus.). NUFIP1 knockdown suppressed cell growth in the colorectal cancer cell lines HT-29 and HCT116 (**Figure 3C**; *P < 0.05 *vs.* sh-Ctrl lentivirus.). Next, we assessed the effect of NUFIP1 knockdown on CRC cell growth *in vivo* in a xenograft nude mouse model. NUFIP1 knockdown significantly reduced tumor volume, signaling and weight in a xenograft nude mouse model (**Figures 3D–F**; *P < 0.05 *vs.* sh-Ctrl lentivirus.).

### NUFIP1 Knockdown Suppresses CRC Cell Proliferation and Induces Cell Apoptosis

**Figure 4:**
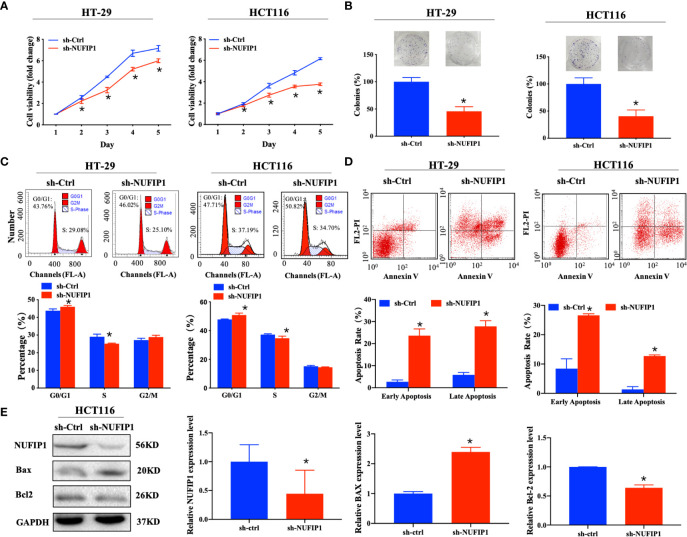
NUFIP1 knockdown inhibits cell proliferation and induces cell apoptosis in colorectal cancer (CRC) cells. HT-29 and HCT116 cells were transduced with lentivirus encoding either anti-NUFIP1 small hairpin RNA (sh-NUFIP1) or control shRNA (sh-Ctrl). **(A)** Cell viability of NUFIP1 knockdown by the CCK-8 assay. Data were normalized to the viability on Day 1 and are represented as fold change. *P < 0.05 *vs.* sh-Ctrl lentivirus. **(B)** Cell survival in colony formation assays. Data were normalized to the survival of control cells. *P < 0.05 *vs.* sh-Ctrl lentivirus. **(C)** Effect of NUFIP1 knockdown on cell cycle progression in HT-29 and HCT116 cells, as measured by flow cytometry. Representative plots showing the distribution of cells in G0, G1, and S phase. *P < 0.05 *vs.* sh-Ctrl lentivirus. **(D)** Effect of NUFIP1 knockdown on apoptosis in HT-29 and HCT116 cells was measured by flow cytometry. Representative plots and quantification of distributions of cells in different stages of apoptosis. *P < 0.05 *vs.* sh-Ctrl lentivirus. **(E)** Bax and Bcl-2 protein expression in HCT116 cells after NUFIP1 knockdown by western blot. Bax and Bcl-2 bands were quantitated using ImageJ software and normalized to GAPDH. *P < 0.05 *vs.* sh-Ctrl group. All experiments were performed in triplicate.

We further explored the effects of NUFIP1 knockdown on cell proliferation and apoptosis in CRC cells. CCK-8 and colony formation assays demonstrated that NUFIP1 knockdown suppressed cell viability (**Figure 4A**; *P < 0.05 *vs.* sh-Ctrl lentivirus.) and survival (**Figure 4B**; *P < 0.05 *vs.* sh-Ctrl lentivirus.) of HT-29 and HCT116 cells. NUFIP1 knockdown also induced cell cycle arrest at G0/G1 (**Figure 4C**; *P < 0.05 *vs.* sh-Ctrl lentivirus.) and apoptosis in HT-29 and HCT116 cells (**Figure 4D**; *P < 0.05 *vs.* sh-Ctrl lentivirus.). Moreover, western blot analysis revealed up-regulation of Bax and down-regulation of Bcl-2 on the protein levels (**Figure 4E**; *P < 0.05 *vs.* sh-Ctrl group.).

### UA Treatment Reinforces the Ability of NUFIP1 Knockdown to Suppress Cell Proliferation and Induce Apoptosis in CRC Cells

**Figure 5:**
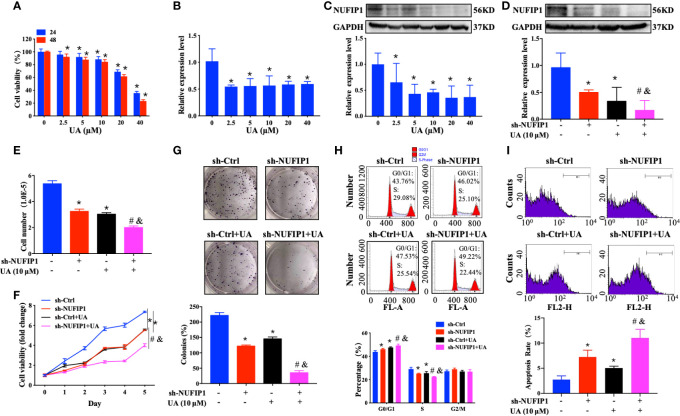
Ursolic acid (UA) inhibits colorectal cancer (CRC) cell growth and regulates NUFIP1 expression. Effect of UA on the expression of HT-29 cells. **(A)** Cell viability of HT-29 cells after treatment with 0, 2.5, 5, 10, 20, 40 µM UA for 24 h or 48 h analyzed by CCK-8 assay. *P < 0.05 *vs.* 0 µM UA group. **(B)** NUFIP1 mRNA level of HT-29 cells after treatment with 0, 2.5, 5, 10, 20, or 40 µM UA for 48 h by qPCR. GAPDH was used as an internal control. *P < 0.05 *vs.* 0 µM UA group. **(C)** NUFIP1 protein level in HT-29 cells after treatment with 0, 2.5, 5, 10, 20, or 40 µM UA for 48 h by western blot. NUFIP1 bands were quantitated using ImageJ software and normalized to GAPDH. *P < 0.05 *vs.* 0 µM UA group. HT-29 cells transduced with lentivirus encoding either anti-NUFIP1 small hairpin RNA (sh-NUFIP1) or control shRNA (sh-Ctrl) were treated with 10 µM UA for 48 h. **(D)** NUFIP1 protein level in HT-29 cells after NUFIP1 knockdown and treatment with 10 µM UA for 48 h, analyzed by western-blot. NUFIP1 bands were quantitated using ImageJ software and normalized to GAPDH. *P < 0.05 *vs.* sh-Ctrl group. #P < 0.05 *vs.* sh-Ctrl + UA group. &P < 0.05 *vs.* sh-NUFIP1 group. **(E)** Growth of HT-29 cells after NUFIP1 knockdown and treatment with 10 µM UA for 48 h by cell counting. *P < 0.05 *vs.* sh-Ctrl group. #P < 0.05 *vs.* sh-Ctrl + UA group. &P < 0.05 *vs.* sh-NUFIP1 group. **(F)** Cell viability of HT-29 cells after NUFIP1 knockdown and treatment with 10 µM UA for 48 h analyzed by CCK-8 assay. Data were normalized to the viability of untreated control cells. *P < 0.05 *vs.* sh-Ctrl group. #P < 0.05 *vs.* sh-Ctrl + UA group. &P < 0.05 *vs.* sh-NUFIP1 group. **(G)** Cell survival of HT-29 cells after NUFIP1 knockdown and treatment with 10 µM UA for 48 h, analyzed by colony formation assay. Data were normalized to the survival of untreated control cells. *P < 0.05 *vs.* sh-Ctrl group. #P < 0.05 *vs.* sh-Ctrl + UA group. &P < 0.05 *vs.* sh-NUFIP1 group. **(H)** Cell cycle in HT-29 cells after NUFIP1 knockdown and treatment with 10 µM UA for 48 h, as analyzed by flow cytometry. Representative plots showing distribution of cells in G0, G1, and S phase. *P < 0.05 *vs.* sh-Ctrl group. #P < 0.05 *vs.* sh-Ctrl + UA group. &P < 0.05 *vs.* sh-NUFIP1 group. **(I)** Apoptosis of HT-29 cells analyzed by annexin V staining, followed by flow cytometry. Representative plots showing the percentages of apoptotic cells. *P < 0.05 *vs.* sh-Ctrl group. ^#^P < 0.05 *vs.* sh-Ctrl + UA group. &*P* < 0.05 *vs.* sh-NUFIP1 group. All experiments were performed in triplicate.

UA exhibits anti-CRC activity by regulating multiple signaling pathways (23, 24). However, its effect on NUFIP1 remained unknown. To explore the potential of NUFIP1 as a therapeutic target in CRC, we investigated the effects of UA treatment on cell growth in HT-29 cells in which NUFIP1 had been knocked down. Interestingly, we found that UA treatment decreased cell viability and down-regulated NUFIP1 expression on both mRNA and protein levels (**Figures 5A–C**; *P < 0.05 *vs.* 0 µM UA group.). Furthermore, UA treatment further down-regulated NUFIP1protein expression (**Figure 5D**; *P < 0.05 *vs.* sh-Ctrl group. #P < 0.05 *vs.* sh-Ctrl + UA group. &P < 0.05 *vs.* sh-NUFIP1 group.), decreased cell number (**Figure 5E**; *P < 0.05 *vs.* sh-Ctrl group. #P < 0.05 *vs.* sh-Ctrl + UA group. &P < 0.05 *vs.* sh-NUFIP1 group.), cell viability (**Figure 5F**; *P < 0.05 *vs.* sh-Ctrl group. #P < 0.05 *vs.* sh-Ctrl + UA group. &P < 0.05 *vs.* sh-NUFIP1 group.), and cell survival (**Figure 5G**; *P < 0.05 *vs.* sh-Ctrl group. #P < 0.05 *vs.* sh-Ctrl + UA group. &P < 0.05 *vs.* sh-NUFIP1 group.). UA also induced cell cycle arrest at G0/G1 (**Figure 5H**; *P < 0.05 *vs.* sh-Ctrl group. #P < 0.05 *vs.* sh-Ctrl + UA group. &P < 0.05 *vs.* sh-NUFIP1 group.) and apoptosis (**Figure 5I**; *P < 0.05 *vs.* sh-Ctrl group. #P < 0.05 *vs.* sh-Ctrl + UA group. &P < 0.05 *vs.* sh-NUFIP1 group.) in CRC cells.

### NUFIP1 Knockdown Induces CRC Cell Senescence Through the HMGA2/SAHF Pathway

**Figure 6:**
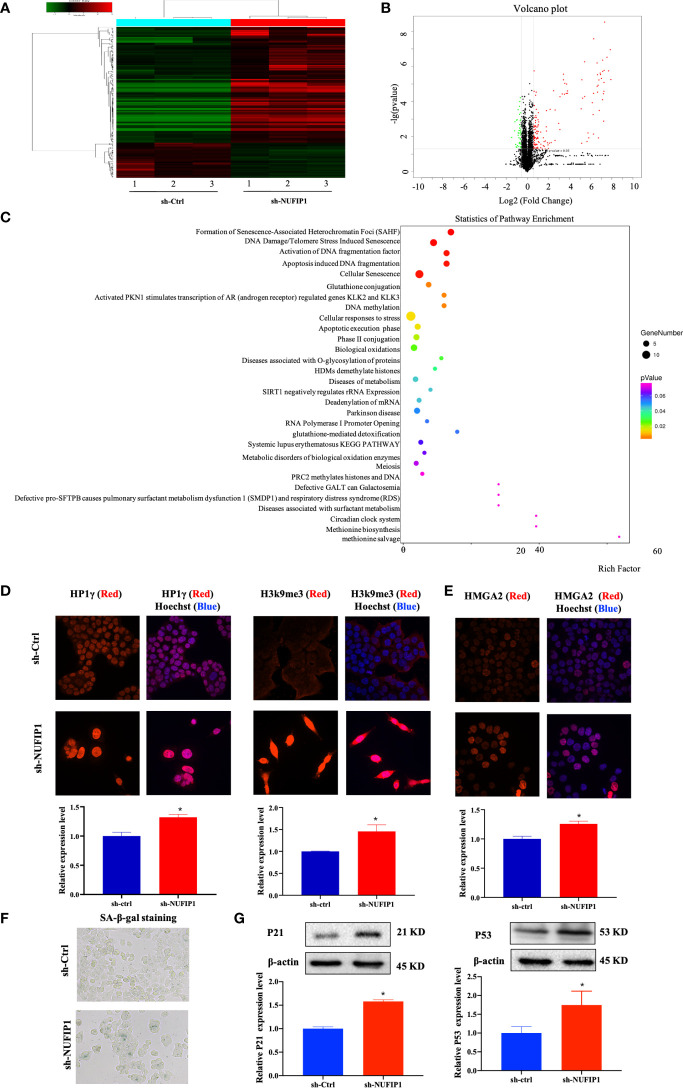
NUFIP1 knockdown induces senescence and formation of senescence-associated heterochromatin foci (SAHF) in colorectal cancer (CRC) cells. HT-29 cells were transduced with lentivirus encoding either anti-NUFIP1 shRNA (sh-NUFIP1) or control shRNA (sh-Ctrl), and the iTRAQ method was used to identify differentially expressed proteins in CRC. A hierarchical clustering plot **(A)** and a volcano plot **(B)** were used to identify different expression of genes (fold change ≥ 1.5, P < 0.05). **(C)** KEGG pathway enrichment analysis was performed to identify functionally related gene pathways based on the Reactome database. The top 10 enriched signaling pathways are shown. **(D)** Immunofluorescence images showing co-localization of NUFIP1 in chromatin foci with the SAHF markers HP1γ and H3k9me3 in HCT116 cells after NUFIP1 knockdown. Representative images were taken at magnification of 400×. *P < 0.05 *vs.* sh-Ctrl group. **(E)** Fluorescent images of HCT116 cells showing the expression of HMGA2 after NUFIP1 knockdown. Representative images were taken at magnification of 400×. *P < 0.05 *vs.* sh-Ctrl group. **(F)** Senescence-associated β-galactosidase staining in HCT116 cells after NUFIP1 knockdown. Representative images were taken at magnification of 400 ×. **(G)** Both p53 and p21protein expression in HCT116 cells after NUFIP1 knockdown was determined by western blot. Both p53 and p21 bands were quantitated using ImageJ software and normalized to β-actin. *P < 0.05 *vs.* sh-Ctrl group.

To further investigate the underlying mechanism of NUFIP1 knockdown on tumor growth suppression in CRC, the isobaric tag for relative and absolute quantitation (iTRAQ) methodology was applied to identify differentially expressed proteins (DEPs) in CRC cells after NUFIP1 knockdown. As shown in **Figures 6A, B**, a total of 177 DEPs were identified, including 136 overexpressed proteins and 41 underexpressed proteins (fold change ≥ 1.5, P < 0.05). A hierarchical clustering plot (**Figure 6A**) and a volcano plot (**Figure 6B**) were used to identify DEPs, and analysis of Kyoto Encyclopedia of Genes and Genomes (KEGG) pathways showed them to be enriched in multiple signaling pathways (**Figure 6C**).

The most enriched signaling pathway was senescence-associated heterochromatin foci (SAHF). Therefore, we turned our focus to the potential effects of NUFIP1 knockdown on cell senescence. First, we used immunofluorescence to detect the SAHF markers HP1γ and H3k9me3 (23, 24), as well as the senescence-related protein HMGA2 (23, 25) in HCT116 cells expressing normal levels of endogenous NUFIP1. Interestingly, we observed low or without HP1γ and H3k9me3 expression in sh-Ctrl cell, while observed both up-regulation HP1γ and H3k9me3 expression, which co-localized with Hoechst-dense chromatin foci (**Figure 6D**; *P < 0.05 *vs.* sh-Ctrl group.). Moreover, when NUFIP1 was knocked down, HMGA2 expression was up-regulated (**Figure 6E**; *P < 0.05 *vs.* sh-Ctrl group.) and β-galactosidase activity was increased (**Figure 6F**), suggesting that NUFIP1 knockdown obviously induced senescence and increased both HMGA2 expression and SAHF. Furthermore, NUFIP1 knockdown significantly up-regulated p53 and its down-stream p21 protein expression (**Figure 6G**; *P < 0.05 *vs.* sh-Ctrl group.).

## Discussion

The key finding of the current study is the identification of NUFIP1 as a potential oncogene in CRC. In our work, overexpression of NUFIP1 in CRC tissues correlated with shorter survival and more advanced cancer stage. Knockdown of NUFIP1 significantly suppressed CRC tumor growth both *in vivo* and *in vitro* by suppressing cell proliferation and inducing cell apoptosis. UA treatment enhanced the anti-tumor effects of NUFIP1 knockdown, suggesting the potential of NUFIP1 as a diagnostic and prognostic marker, as well as therapeutic target in CRC. NUFIP1 knockdown induced cell senescence and activated both by activating the HMGA2/SAHF and p53/p21signaling pathways, suggesting a novel strategy for anti-CRC treatment. These observations lead us to propose an oncogenic NUFIP1/HMGA2/SAHF axis.

DEG screening and shRNA-based validation identified multiple potential oncogenes in CRC, including NUFIP1. Few studies have examined a role for NUFIP1 in cancer, which encouraged us to assess its potential involvement in CRC. Moreover, both mRNA and protein levels of NUFIP1 were increased in CRC tissues, and higher expression of NUFIP1 correlated with worse prognosis and CRC progression. This suggests the potential of NUFIP1 as an early diagnostic and prognostic marker for CRC. These findings should be verified and extended in a much larger clinical sample.

The biological function of NUFIP1 in CRC had never been explored, our current study for the first time revealed that NUFIP1 knockdown significantly suppressed CRC cell growth both *in vitro* and *in vivo* by inhibiting cell proliferation and inducing cell apoptosis, while NUFIP1 overexpression had a minor effect on promoting cell proliferation in Caco2 cell (unpublished data), suggesting the essential role of NUFIP1 on tumor growth. However, NUFIP1 exhibits a minor effect on cell cycle arrest at G0/G1 phase, while significantly induces cell apoptosis and cell senescence of CRC cells. Moreover, our current study found that UA further enhanced the anti-tumor effects of NUFIP1 knockdown for the first time. These data indicate that NUFIP1 regulates tumor growth, suggesting that it may serve as a therapeutic target in CRC. Our results with UA further suggest that developing selective small-molecule inhibitors of NUFIP1 may provide a promising therapeutic strategy against CRC.

Previous studies indicated that NUFIP1 participates in multiple biological processes, including snoRNP biogenesis (10–13), mRNA export and localization, and transcription (15). DEP screening in HCT116 cells after NUFIP1 knockdown identified 136 overexpressed and 41 underexpressed proteins, with several of those DEPs (including E2F3, STAT1) involved in regulating tumor growth (26, 27). Surprisingly, SAHF was the most enriched signaling pathways among the DEPs, which we confirmed by showing that NUFIP1 knockdown in HCT116 cells increased β-galactosidase activity, levels of SAHF markers HP1γ and H3k9me3 in the nuclei, and levels of HMGA2. Moreover, chronic activation of p53/p21 pathway play an essential role during the process of cellular senescence (28). Our current study revealed that NUFIP1 knockdown up-regulated the protein expression of both p53 and p21 pathway. Our results suggest that activation of the HMGA2/SAHF signaling pathway and p53/p21 pathways might be one of essential mechanisms contributing to senescence of CRC after NUFIP1 knockdown. However, the role of NUFIP1 on the senescence of CRC cells and its underlying mechanisms should be further addressed in future study. Moreover, future work should address the regulatory effects of NUFIP1 on cell proliferation and apoptosis, as well as on other DEPs and enriched signaling pathways.

Our analyses of patient materials, cell lines and tumor xenografts suggest that NUFIP1 is an oncogene that drives tumor growth and progression in CRC. It may be a useful diagnostic or prognostic marker for the disease. The ability of UA to counteract the oncogenic effects of NUFIP1 in CRC suggests that the protein may be a good therapeutic target. Future work should further explore the interaction between UA and NUFIP1.

## Data Availability Statement

The original contributions presented in the study are included in the article/**Supplementary Material**. Further inquiries can be directed to the corresponding author.

## Ethics Statement

Collection of the clinic samples and related experiments were approved by the Ethics Committee of FJTCM. Written, informed consent was obtained from all patients or their families. All animal maintenance and procedures were performed in strict accordance with the “Guide for the Care and Use of Laboratory Animals” and the “Principles for the Utilization and Care of Vertebrate Animals” and approved by the Animal Committee of FJTCM.

## Author Contributions

LL, JP, and FC conceived and designed the experiments. LL, AS, LYL, and MZ conducted bioinformatics analysis and IHC-based TMA analysis. MW, LYL, YQC, MZ, QX, and XZ performed cell cultures, CCK-8 assays and colony formation assays. YQC, MZ, and LYL conducted cell cycle analysis. MW, AS, YC, and XC performed the animal experiments and data analysis. NX, JC, and LW conducted western blotting and data analysis. AS, JL, and LYL conducted pathway analysis and verification. MW and AS wrote the manuscript, which TS, LL, and JP revised. All authors contributed to the article and approved the submitted version.

## Funding

This work was supported by the National Natural Science Foundation of China (grants 81803882, 81673721, and 81703913), the International Cooperative Project of Fujian Department of Science and Technology (2017I0007), and the Natural Science Foundation of Fujian Province (2017J01846).

## Conflict of Interest

The authors declare that the research was conducted in the absence of any commercial or financial relationships that could be construed as a potential conflict of interest.

## Publisher’s Note

All claims expressed in this article are solely those of the authors and do not necessarily represent those of their affiliated organizations, or those of the publisher, the editors and the reviewers. Any product that may be evaluated in this article, or claim that may be made by its manufacturer, is not guaranteed or endorsed by the publisher.
