# Supplementary material for: Targeting NUFIP1 Suppresses Growth and Induces Senescence of Colorectal Cancer Cells
Source: Front Oncol. 2021 Jul 23;11:681425. doi: 10.3389/fonc.2021.681425 (PMC8343530; doi:10.3389/fonc.2021.681425)
Supplement: Supplementary file 1 [file Table_1.docx]

Supplementary Material

# Supplementary Table S1

Differences in the expression of 10 selected genes between colorectal cancer (CRC) tissues and noncancerous colorectal tissues.

| **Gene symbol** | **Fold change^1^** |
| --- | --- |
| NUFIP1 | 3.35 |
| FAM92A1 | 3.87 |
| NEBL | 4.72 |
| PLEKHS1 | 3.66 |
| PRPF4 | 3.00 |
| CGREF1 | 4.81 |
| POLR1B | 2.72 |
| HILPDA | 4.45 |
| TAF1D | 2.36 |
| NUDCD1 | 3.21 |

1 All P-values < 0.001.

# Supplementary Table S2

Sequences of primers used in this study.

| **Name** | | **Primers (5‘-3’)** |
| --- | --- | --- |
| NUFIP1  GAPDH | F:5’-CCAGGTTCTTGATAGCAGTGC-3’ R:5’-GATGGTGTGTTCTTGGTTCG-3’  F :5’- ATGGGGAAGGTGAAGGTCG-3’  R :5’-GGGGTCATTGATGGCAACAATA-3’ | |

# Supplementary Table S3

Clinic pathological features of 80 CRC patients

| Characteristic | | N（%） |
| --- | --- | --- |
| Age（years） | <65 | 44 (55) |
|  | ≥ 65 | 36 (45) |
| Gender | Female | 33 (41.25) |
|  | Male | 47 (58.75) |
| Clinical stage | Ⅰ | 3 (3.75) |
|  | Ⅱ | 58 (72.5) |
|  | Ⅲ | 19 (23.75) |
| Lymph node metastasis |  | 28 (35) |

| Characteristic | | n (%) |
| --- | --- | --- |
| Age (years) | < 65 | 35 (37) |
|  | ≥ 65 | 58 (62) |
| Gender | Male | 51 (54) |
|  | Female | 42 (45) |
| Tumor size | < 5cm | 46 (49) |
|  | ≥ 5cm | 46 (49) |
| Clinical stage | Ⅰ+ Ⅱ | 49 (52) |
|  | Ⅲ + Ⅳ | 45 (48) |
| T stage | T1+T2 | 6 (6) |
|  | T3+T4 | 84 (89) |
| N stage | N0 | 56 (60) |
|  | N1 | 28 (30) |
|  | N2 | 9 (9) |
| M stage | M0 | 91 (97) |
|  | M1 | 3 (3) |
| Distant metastasis |  | 0 (0) |

**Supplementary Table S4.**

Clinic pathological features of 94 CRC patients
